# Endothelial progenitor cells systemic administration alleviates multi-organ senescence by down-regulating USP7/p300 pathway in chronic obstructive pulmonary disease

**DOI:** 10.1186/s12967-023-04735-x

**Published:** 2023-12-06

**Authors:** Wenhua Wang, Huaihuai Peng, Menghao Zeng, Jie Liu, Guibin Liang, Zhihui He

**Affiliations:** 1 Department of Intensive Care Unit, The Third Xiangya Hospital, Central South University, Changsha, Hunan China; 2 Department of Intensive Care Unit, Hunan Province Directly Affiliated Traditional Chinese Medicine Hospital, Zhuzhou, Hunan China

**Keywords:** COPD, Senescence, EPCs, Systemic administration, USP7, p300

## Abstract

**Background:**

Chronic obstructive pulmonary disease (COPD) has impacted approximately 390 million people worldwide and the morbidity is increasing every year. However, due to the poor treatment efficacy of COPD, exploring novel treatment has become the hotpot of study on COPD. Endothelial progenitor cells (EPCs) aging is a possible molecular way for COPD development. We aimed to explore the effector whether intravenous administration of EPCs has therapeutic effects in COPD mice.

**Methods:**

COPD mice model was induced by cigarette smoke exposure and EPCs were injected intravenously to investigate their effects on COPD mice. At day 127, heart, liver, spleen, lung and kidney tissues of mice were harvested. The histological effects of EPCs intervention on multiple organs of COPD mice were detected by morphology assay. Quantitative real-time PCR and Western blotting were used to detect the effect of EPCs intervention on the expression of multi-organ senescence-related indicators. And we explored the effect of EPCs systematically intervening on senescence-related USP7/p300 pathway.

**Results:**

Compared with COPD group, senescence-associated β-galactosidase activity was decreased, protein and mRNA expression of p16 was down-regulated, while protein and mRNA expression of cyclin D1 and TERT were up-regulated of multiple organs, including lung, heart, liver, spleen and kidney in COPD mice after EPCs system intervention. But the morphological alterations of the tissues described above in COPD mice failed to be reversed. Mechanistically, EPCs systemic administration inhibited the expression of mRNA and protein of USP7 and p300 in multiple organs of COPD mice, exerting therapeutic effects.

**Conclusions:**

EPCs administration significantly inhibited the senescence of multiple organs in COPD mice via down-regulating USP7/p300 pathway, which presents a possibility of EPCs therapy for COPD.

**Supplementary Information:**

The online version contains supplementary material available at 10.1186/s12967-023-04735-x.

## Background

Chronic obstructive pulmonary disease (COPD) is a continuously progressive, incompletely reversible airway obstructive disease with high morbidity and mortality, contributing to social burden [1]. Statistically, 390 million people worldwide suffered from COPD up to 2019, and 80.5% of patients in middle and low-income countries. The incidence of COPD has been on the rise with increasing smoking rates and aging population in many countries [2]. COPD is currently the third most common reason of death worldwide, accounting for 5.8% in total disease-related mortality [3]. Because the molecular mechanisms of COPD pathogenesis are not fully understood, the current treatment strategy for COPD is to control symptoms and slow down the disease progression. Although symptoms are alleviated for patients, the recurrence is common after drug withdrawal [4]. Therefore, exploring the deeper molecular mechanisms of COPD pathogenesis can facilitate the discovery of novel treatments, and this has become an important direction of COPD investigation at present. Currently, constrained by ethics, most studies on the pathogenesis and treatment of COPD are conducted using experimental animal models, mice being the most appropriate choice [5]. The mouse lung’s structure and physiological processes are similar to those of humans, and mice COPD model accurately depicts important features of COPD patients, such as predisposing factors and lung dysfunction [6]. Moreover, COPD is a systemic disease characterized by persistent chronic pulmonary and systemic inflammation, which is considered to be the driving factor for disease initiation and progression [7]. Therefore, animal models are more accurate in reflecting the entire state of the disease and recapitulating the complex, multi-system pathology of the disease. As 80% of COPD patients have a smoking etiology, cigarette smoke (CS) serves as a pivotal stimulus for COPD modeling in mice, and the respiratory responses to CS are similar in mice and humans [7, 8]. Our study used CS exposure-induced COPD mice for the experiments.

Notably, the morbidity of COPD grows exponentially with age, revealing a close relationship between aging and COPD. CS exposure triggers oxidative stress and inflammation, leading to alveolar senescence in COPD mice, exacerbating emphysema [9, 10]. CS exposure-induced COPD mice suffer from mitochondrial damage, accompanied by accelerated cellular senescence [11]. Studies showed that the expression of senescence-related proteins was increased in the lungs and peripheral circulation of COPD patients [12]. The telomere lengths of alveolar type II epithelial cells, endothelial cells and circulating leukocytes were significantly shorter in COPD patients than in controls [13, 14]. These results suggest that aging is a potential molecular way for the progression of COPD. Furthermore, our team extended the relationship between COPD and aging to organs besides lung for the first time. It was shown that senescence existed in multiple organs, including heart, liver, spleen and kidney in COPD mice [15]. Normally, aging cells are renewed by stem cells. Therefore, the aging of stem cells can impact the systemic aging.

Stem cells are the class of potential self-renewing and multi-directional differentiated with powerful tissue repair capacity. Endothelial progenitor cells (EPCs), a group of adult stem cells, are precursor cells of endothelial cells and play essential roles in angiogenesis, vascular regeneration and repair [16]. It has been demonstrated the presence of resident EPCs in the vessel wall and lung tissue, and these EPCs are involved in repair of injured tissue [17, 18]. Multiple studies have shown that the decreased circulating EPCs with dysfunction in COPD patients failed to repair damaged vascular endothelial cells and may be involved in the development of COPD [19, 20]. Therefore, intervening senescent EPCs is promising for the treatment of COPD. Stem cell therapy is an emerging therapy to repair and regenerate damaged cells and tissues by transplanting healthy stem/progenitor cells into patients [21]. Stem/progenitor cell therapy is now increasingly becoming an effective treatment for many diseases, such as myocardial infarction, chronic trauma, and lung injury [22–24]. Common stem/progenitor cells for COPD treatment are mesenchymal stromal stem cells (MSCs) [25]. Up to date, clinical trials with MSCs have been demonstrated to be safety in COPD patients. Yet no changes were found in lung function tests or outcomes of quality life, and larger clinical trials are still required to prove the effectiveness of MSCs in COPD [26]. EPCs were known as dysfunctional in COPD patients, so we want to explore whether EPCs therapy could be useful in the treatment of COPD, aiming to provide more possibilities for stem cell therapy in COPD.

In this experiment, simulating clinical COPD patients, we constructed CS-induced COPD mice models and treated systemically with EPCs intravenous injection. At the end of treatment, we examined morphological alterations in lung, heart, liver, spleen and kidney tissues as well as the expression of aging-related indicators to clarify whether EPCs transplantation could treat multi-organ aging in COPD mice and further explore the mechanisms involved.

## Methods

### EPCs isolation and characterization

Six-week-old C57BL/6 J male mice were enrolled. Animals were killed by overdose of anesthetic. The shoulder, hip and ankle joints were bluntly separated in cleanbench, and the long bones of the upper and lower limbs were completely separated, the attached muscle tissues were fully stripped off, and then the joints at both ends were cut to expose the bone marrow cavity. Bone marrow cavity was fully flushed with Medium 199 (M199, Thermo, 11043023). The flushing solution was gathered in the centrifugal tube and mixed well. The washing solution was slowly added to the centrifuge tube containing the equal volume of lymphocyte separation solution (Sigma, 10,771), centrifuged with 2500 rpm for 30 min (min). Then the intermediate layer was sucked out and washed with M199, and centrifuged with 1500 rpm for 10 min. The cells were resuscitated in 1 ml EGM-2 medium (Lonza, CC-3156) and counted. Cells (5–10 × 10^6^ per well) were seeded in 6-well plate and added 2 ml EGM-2 medium to each well, cultured in 37 ℃, 5% CO_2_ incubator. The first full amount of medium exchange was performed on the 4th day of culture, followed by half volume medium changed every 3–4 days for subsequent animal experiments. EPCs were identified using the dual fluorescent labeling method, DiI-Ac-LDL (red) and FITC-UAE-1 (green), before import into mice (Additional file 1: Figure S1).

### Animal experiments

All animal experiments were performed with 6-week specific-pathogen-free male mice. C57BL/6 J mice were purchased from Hunan SJA Laboratory Animal Co., Ltd. All animals were placed in a 12-h light/12-h dark cycle and SPF conditions under constant temperature and humidity control in the Department of Experimental Animals, Central South University. All studies were performed following the experimental protocols approved by the Ethics Committee of Central South University.

### Establishment and evaluation of COPD mice

The mice were placed in a smoke box (69 cm × 47 cm × 38 cm) and five cigarettes were burned simultaneously each time, which lasted for 15 min, and then the glass box was opened to allow the mice to break for 5 min, followed by a repeat burning of five cigarettes for 15 min. This procedure is described as a cycle of CS exposure. The mice were exposed for 2 cycles per day with a 20 min interval between two cycles, 6 days per week for 16 weeks. Evaluating whether the COPD model is successfully constructed according to mean lung interlining interval (MLI) and destruction index (DI), details were followed previously described [27].

### The treatment of COPD mice and harvest of tissues

C57BL/6 J mice were divided into four groups: control, COPD, COPD+EPCs, COPD+P5091 (n = 10). The corresponding mice were injected intravenously with EPCs (1*10^6^/20 *g*) [28, 29] or phosphate buffered saline (PBS, 100 μl/mouse) on days 90, 105 and 120, or ubiquitin specific protease 7 (USP7) inhibitor P5091 (5 mg/kg, Selleck, S7132) or PBS (100 μl/mouse) once every 3 days from 90 to 120 days during modeling, respectively. In the terminal point (on day 127), lung, heart, liver, spleen, and kidney tissues of experimental mice were obtained intactly, washed with PBS, and placed in enzyme-free cryogenic vials and tubes containing 4% paraformaldehyde (Biosharp, BL539A) to be stored at – 80 ℃ and paraffin-embedded, separately.

### Hematoxylin–eosin (HE) staining

For dewaxing, the tissue slices were serially placed into dimethylbenzene (HUSHI, 10023418) for 10 min twice, ethanol absolute (HUSHI, 10009218) for 5 min twice, 95% alcohol for 5 min, 90% alcohol for 5 min, 80% alcohol for 5 min and 70% alcohol for 5 min and finally washed with distilled water. To stain the nuclei, the slices were sequentially put into hematoxylin dye (Sigma, H3136) for 5 min, acid alcohol differentiation solution (Servicebio, G1039) for 5 s, and 0.6% ammonia (Servicebio, G1040) for 5 s, and each time was flushed with flowing water. The slices were then stained with eosin solution (Sigma, 861006) for 3 min for staining the cytoplasm. The slices were then sequentially placed in 95% alcohol for 5 min twice, ethanol absolute for 5 min twice, and xylene for 5 min twice in order to be dehydrated. The slices were slightly dried and sealed with neutral gum. Finally, the slices were observed under a microscope and images were captured.

### Staining and quantification of Senescence-associated β-galactosidase (SA-gal)

Tissues were wrapped with OTC frozen slice embedding agent (SAKURA, 4583) and sliced to 6-8 µm thickness, patches. After restoring to room temperature, the slices were washed with distilled water for 5 min twice. Each sample was dropped with about 50 μl SA-gal staining fixative (Beyotime, C0602; Servicebio, G1073), incubated in a wet box for 15 min at room temperature and washed with PBS for 5 min thrice. Each sample was added with the proper staining working solution, incubated in a wet box overnight at 37 ℃. Removing the staining working solution, nuclei were counterstained using nuclear fast red staining solution (Servicebio, G1035) for 3 min. The slices were sealed with glycerol, and finally images were captured with microscope.

### Western blotting

The proper tissues were washed twice with pre-cooled PBS and lysed with RIPA buffer (Solarbio, R0010) adding Phenylmethanesulfonyl fluoride in a biological sample homogenizer until fully ground and 15 min on ice. Protein concentrations were measured by Bicinchoninic Acid protein Assay Kit (CWBIO, CW1104S) and adjusted to the same concentration with SDS loading buffer (NCM Biotech, WB2001). Then, the protein was electrophoresed in 10% SDS–polyacrylamide gel and transferred onto PVDF membrane (Millipore, IPVH00010). After blocking with 5% skim milk, the membrane was incubated with primary antibody overnight at 4 ℃ [USP7, 1:1000, abcam, ab108931; p300, 1:1000, abcam, ab275378; cyclin D1, 1:1000, abcam, ab16663; Telomerase reverse transcriptase (TERT), 1:500, Beyotime, AF8115; p16 (INK4a), 1:500, Beyotime, AF6471; β-actin, 1:1000, abcam, ab8226]. After being washed thorough with Tris-buffered-saline with Tween-20 (TBST), the membranes were incubated with secondary antibodies (1:5000, Abclonal, AS003 and AS014) for 1 h at room temperature and washed with TBST for 10 min triple. Immunoblots were detected with the ECL Ultra kit (NCM Biotech, P10300). Image acquisition was performed using Odyssey Fc Imaging System (LI-COR Biosciences, Lincoln) and grayscale quantitative analysis was carried with ImageJ software.

### Quantitative real-time PCR assay (qRT-PCR)

The tissue added with Trizol (Thermo, 15596026) was placed in the homogenizer fully ground, and then total RNA was extracted following the protocol. The cDNA was then retrotranscribed using cDNA Synthesis Kit (CWBIO, CW0741). Each component was added to 20 μl reactive system following the manual and qRT-PCR was conducted using a QuantStudio-3 real-time PCR system (Thermo Fisher Scientific). Data were analyzed using the ΔΔCt method, and relative expression was determined by actin normalization. The primer sequences used in this study are shown in Additional file 2: Table S1.

### Statistical analysis

Plotting was completed in GraphPad Prism 8. Statistical analysis was accomplished with SPSS 26 software. The mean ± standard deviation was applied for the measurement data with normal distribution. Normally distributed data means were compared with one-way Analysis of Variance (ANOVA) analysis in multiple groups and t-test between two groups. Differences were considered statistically significant when *p* < 0.05.

## Results

### EPCs therapy and P5091 failed to reverse the pathological changes of lung morphology in COPD mice

**Fig. 1:**
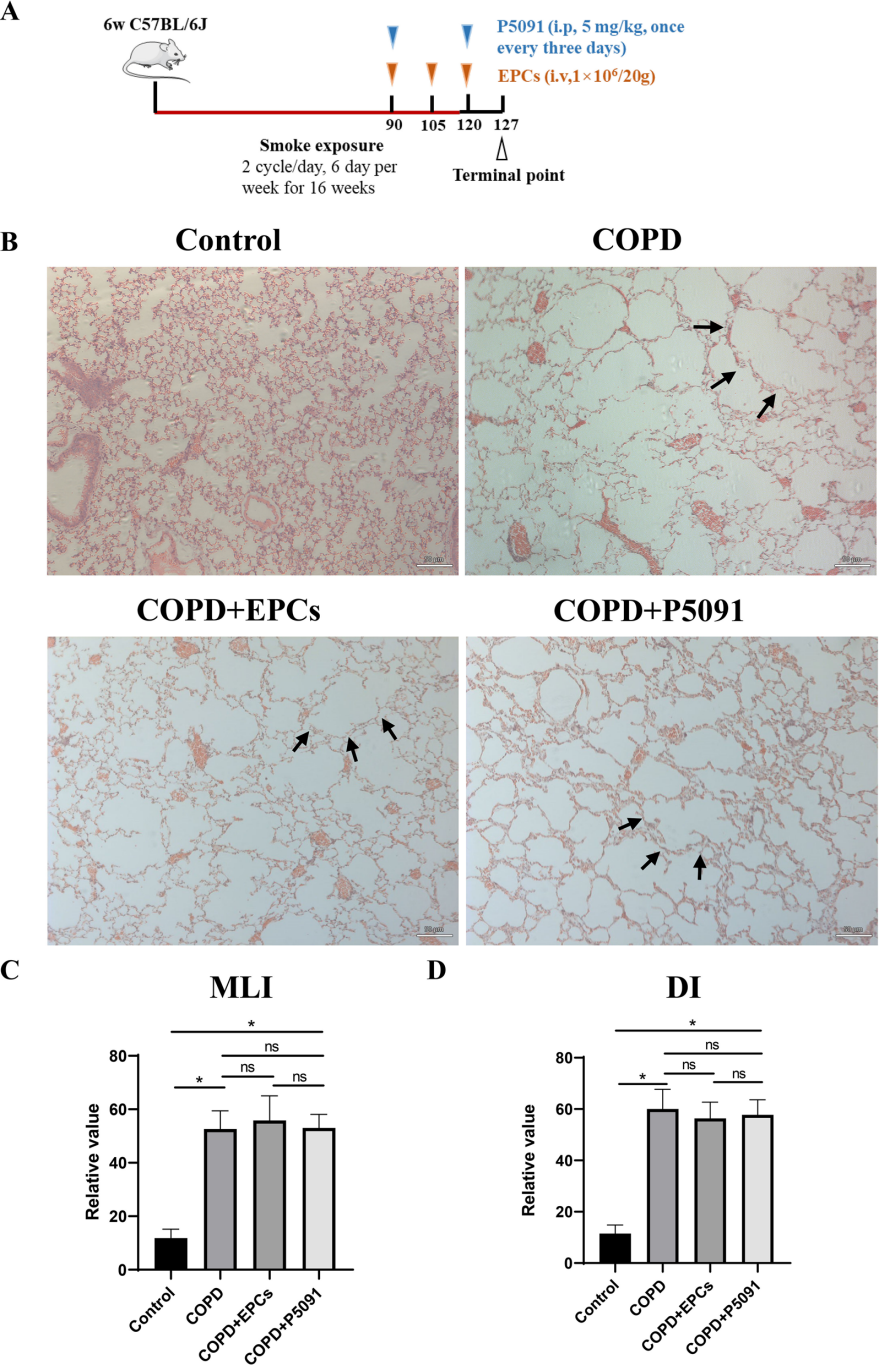
HE staining, MLI and DI score of lung tissue. **A** Experimental protocol of COPD mice modeling and treatment; **B** representative images of HE staining of lung tissue in the control, COPD, COPD+EPCs and COPD+P5091 groups, with a scale of 50 μm; **C** and **D** scores of pathomorphological indexes MLI and DI of lung tissue in the four groups. In **C** and **D**, p values were calculated by one-way ANOVA in multiple groups and two-sided t-test between two groups. *p < 0.05; ns means p > 0.05

Previous studies showed the presence of multi-organ aging in emphysematous mice, including lung, heart, liver, spleen and kidney [15, 30]. The findings indicated the existence of systemic pathological changes in mice with emphysema or COPD, which also exacerbated the severity of COPD and suggested the importance of systemic therapy for COPD patients. To investigate the effect of systemic therapy with EPCs cells on COPD mice, mimicking COPD patients in clinics, we constructed COPD mice models by CS exposure and then intervened them with EPCs cells intravenously and with P5091, the USP7 inhibitor, as a positive control, which has been shown to be effective in senescent cells [31] (Fig. 1A). The results of HE staining showed that compared with control group, in COPD group, COPD+EPCs group, and COPD+P5091 group, the alveoli were significantly dilated, the septa became thinner and fractured, and the dilated alveoli fused to form larger gas-containing cavities (Fig. 1B). However, morphological changes in lung tissue were not ameliorated after EPCs or P5091 treatment. Also, to give more credibility for the results, the pathomorphological indexes MLI and DI were introduced. The results showed that the MLI was 11.83 ± 2.69 μm, 52.37 ± 3.79 μm, 52.83 ± 6.65 μm and 53.03 ± 4.13 μm, as well as the DI was 11.51% ± 2.73%, 60.03% ± 6.29%, 56.38% ± 5.15%, and 57.75% ± 4.82% in normal group, COPD group, COPD+EPCs group, and COPD+P5091 group, respectively (Fig. 1C and D). Consistent with the HE staining, MLI and DI were significantly increased in COPD group, COPD+EPCs group, and COPD+P5091 group when compared with control group (p < 0.05), implying more significant alveolar devastation. However, there was no significant difference in MLI and DI among COPD group, COPD+EPCs group and COPD+P5091 group (p > 0.05). The above results suggested that short-term systemic treatment with EPCs and P5091 could not reverse the pathomorphological changes of lung tissue in COPD mice.

In addition, the morphology of other organs was also investigated. As shown in the Additional file 3: Figure S2, the heart, liver, spleen and kidney tissues of COPD mice showed normal structure, without congestion, hemorrhage, or infiltration of inflammatory cells, and no cell degeneration and necrosis, which was also in accordance with the previous study. Moreover, neither EPCs or P5091 intervention had any effect on the morphological changes of the organs.

### EPCs therapy attenuated aging of lung tissue in COPD mice

**Fig. 2:**
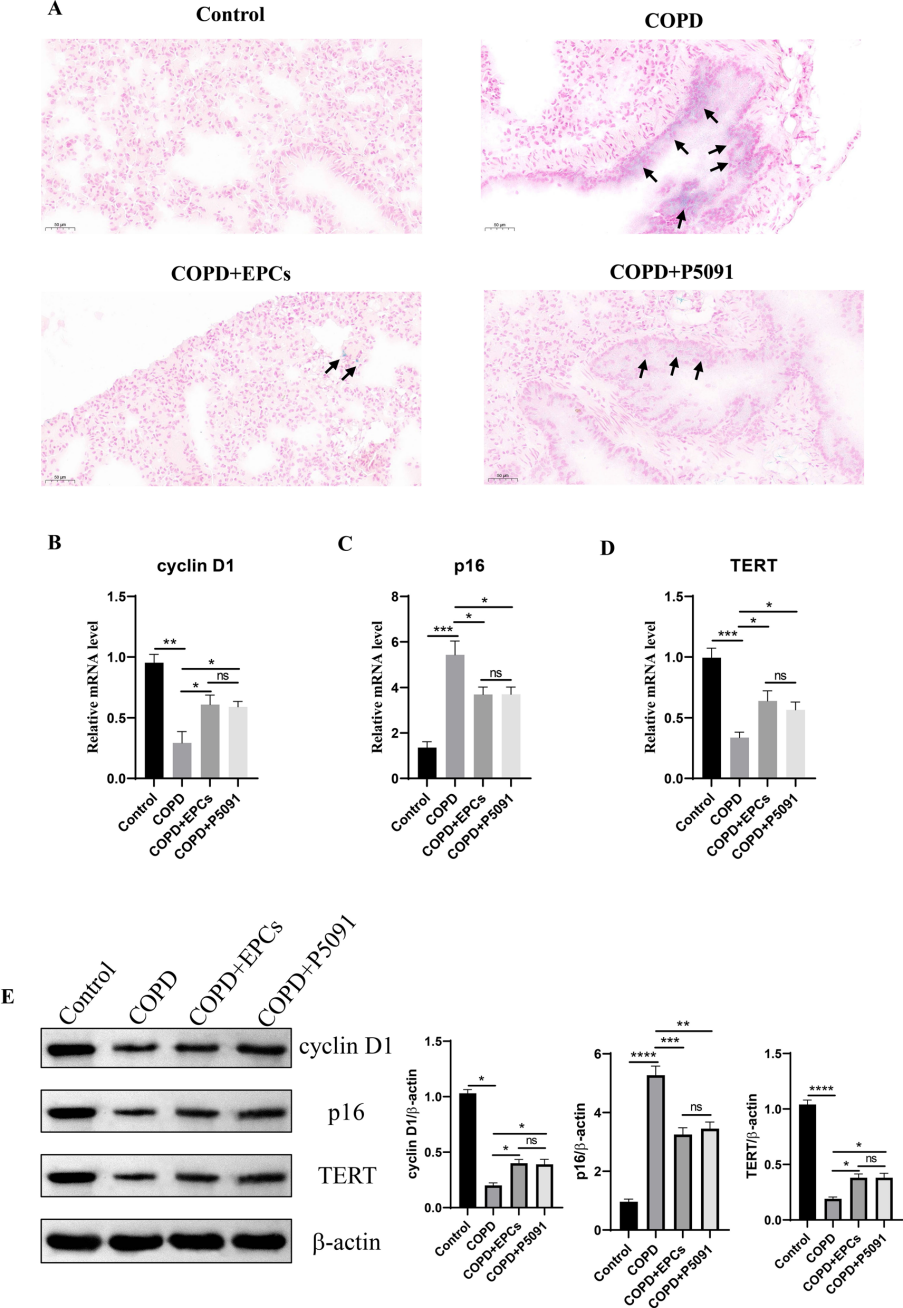
Expression of aging-related indicators in control, COPD, COPD+EPCs and COPD+P5091 groups of lung tissue. **A** Representative pictures of SA-gal staining (blue). Scale indicates 50 μm; **B**–**D** qRT-PCR assay was used to analysis the relative mRNA expression of cyclin D1, p300 and TERT; **E** Western blot was applied to analysis the protein expression of cyclin D1, p300 and TERT. β-actin was used as internal control. p values were calculated by one-way ANOVA in multiple groups and two-sided t-test between two groups. *p < 0.05; **p < 0.01; ***p < 0.001; ****p < 0.0001; ns means p > 0.05

Based on the results of the previous study and Fig. 1, it was shown that although the morphology of lung tissue was not affected by COPD, aging-related markers were altered at the molecular level. Therefore, to investigate whether EPCs cell therapy corrected the changes of aging-related indicators in lung tissues of COPD mice, the activity of SA-gal, a specific marker of cellular aging, was explored in lung tissues. The results showed that compared with control group, COPD, COPD+EPCs and COPD+P5091 groups exhibited increased positivity of SA-gal staining, and the positivity of SA-gal staining of COPD+EPCs and COPD+P5091 groups was significantly less than COPD group (Fig. 2A). Meanwhile, we examined other aging-related parameters in lung tissue, including cyclin D1, p16 (INK4a) and TERT. qRT-PCR results revealed that the mRNA expression of cyclin D1 and TERT was significantly down-regulated (p < 0.01), while the expression of p16 (INK4a) was significantly up-regulated (p < 0.001) in lung tissue of COPD group when compared with control group. Moreover, in COPD+EPCs and COPD+P5091 groups, mRNA expression of cyclin D1 and TERT in lung tissue were elevated when compared with COPD group (p < 0.05), while the expression of p16 (INK4a) was decreased (p < 0.05). mRNA expression of these markers was not significantly different between COPD+EPCs and COPD+P5091 groups **(**Fig. 2B–D**)**. Similar to the trend of qRT-PCR, Western blotting results indicated that the protein expression of cyclin D1 and TERT was significantly down-regulated in COPD group when compared with control group (p < 0.05), while the protein expression of p16 (INK4a) was significantly up-regulated (p < 0.0001). Also, the protein expression of cyclin D1 and TERT in lung tissue was increased in COPD+EPCs and COPD+P5091 groups when compared with COPD group (p < 0.05), while the expression of p16 (INK4a) was decreased (p < 0.01). There was no significant difference in the protein expression of the indicators mentioned above between COPD+EPCs and COPD+P5091 groups (Fig. 2E). These results illustrated that, in terms of transcriptional and translational levels, pro-aging indicators were significantly up-regulated and anti-aging indicators were markedly down-regulated in the lung tissue of COPD mice. And EPCs treatment alleviated the aging severity of lung tissue in COPD mice, and the interference of P5091 made this result more conclusive.

### EPCs cell therapy alleviates multi-tissue senescence

**Fig. 3:**
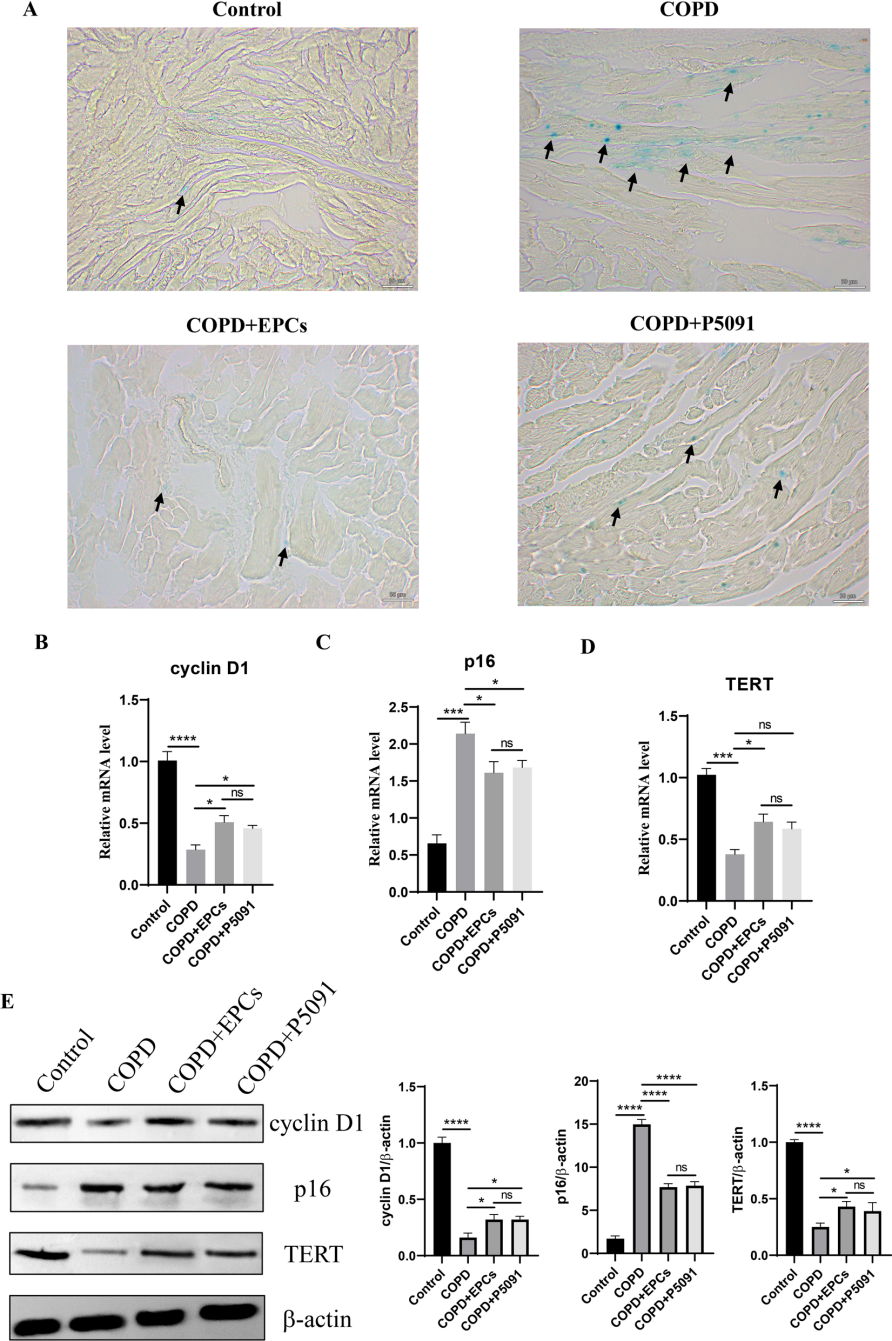
Expression of senescence-related markers in the control, COPD, COPD+EPCs and COPD+P5091 groups of heart tissue. **A** Representative pictures of SA-gal staining (blue). Scale indicates 50 μm; **B**–**D** qRT-PCR experiments was used to analysis the relative mRNA expression of cyclin D1, p300 and TERT; **E** Western blot was used to analysis the protein expression of cyclin D1, p300 and TERT. β-actin was applied as internal control. p values were calculated by one-way ANOVA in multiple groups and two-sided t-test between two groups. *p < 0.05; **p < 0.01; ***p < 0.001; ****p < 0.0001; ns means p > 0.05

**Fig. 4:**
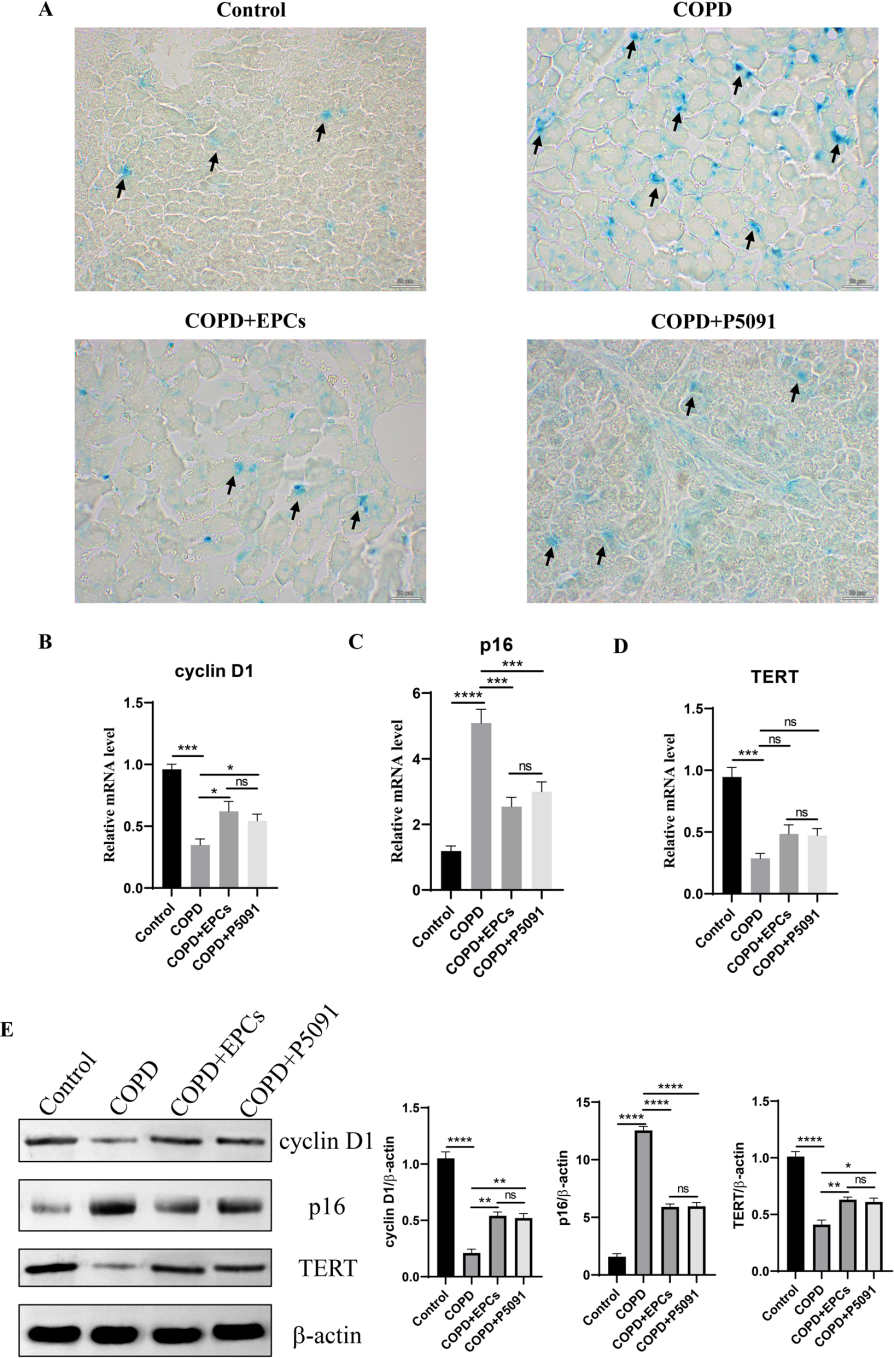
Expression of aging-associated indicators in control, COPD, COPD+EPCs and COPD+P5091 groups of liver tissue. **A** Representative images of SA-gal staining (blue). Scale indicates 50 μm; **B**–**D** qRT-PCR assay was used to analysis the relative mRNA expression of cyclin D1, p300 and TERT; **E** Western blot was served to analysis the protein expression of cyclin D1, p300 and TERT. β-actin was used as internal control. *p* values were calculated by one-way ANOVA in multiple groups and two-sided t-test between two groups. *p < 0.05; **p < 0.01; ***p < 0.001; ****p < 0.0001; ns means* p* > 0.05

**Fig. 5:**
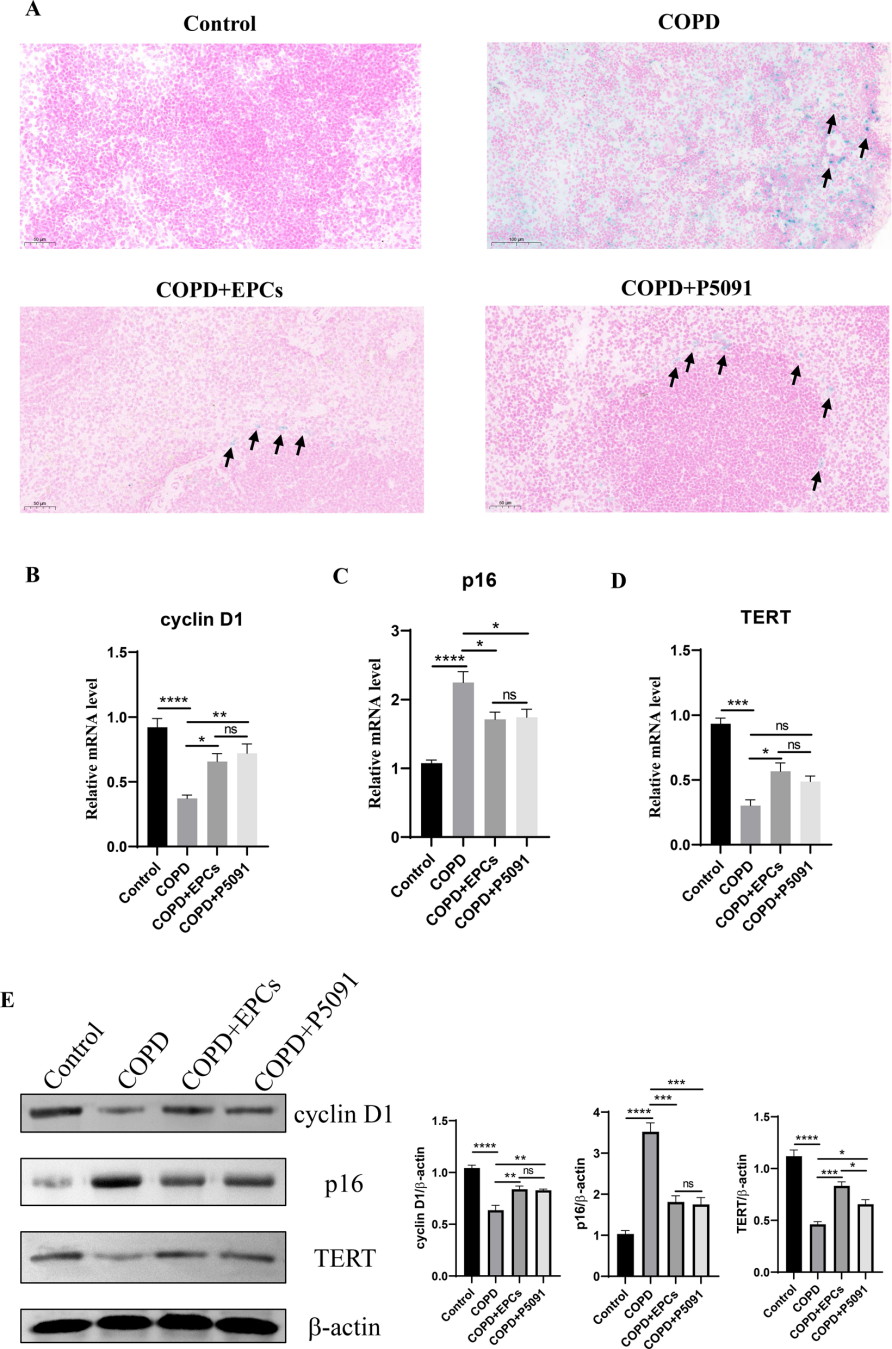
Expression of senescence-associated markers in the control, COPD, COPD+EPCs and COPD+P5091 groups of spleen tissue. **A** Representative pictures of SA-gal staining (blue). Scale indicates 50 μm; **B**–**D** qRT-PCR experiments was used to analysis the relative mRNA expression of cyclin D1, p300 and TERT; **E** Western blot was applied to analysis the protein expression of cyclin D1, p300 and TERT. β-actin was applied as internal control. *p* values were calculated by one-way ANOVA in multiple groups and two-sided t-test between two groups. *p < 0.05; **p < 0.01; ***p < 0.001; ****p < 0.0001; ns means* p* > 0.05

**Fig. 6:**
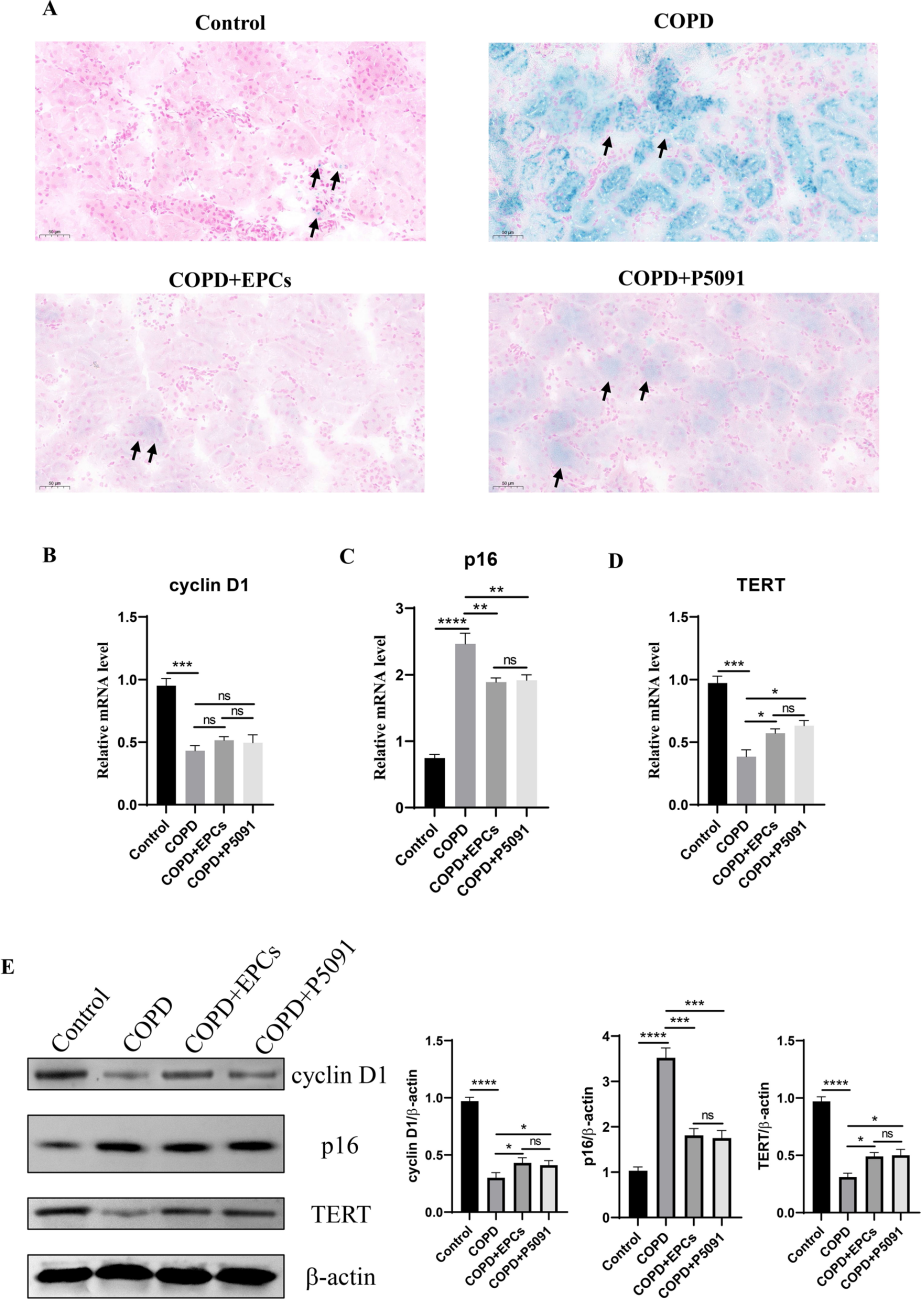
Expression of aging-related indicators in control, COPD, COPD+EPCs and COPD+P5091 groups of kidney tissue. **A** Representative pictures of SA-gal staining (blue). Scale indicates 50 μm; **B**-**D** qRT-PCR assay was used to analysis the relative mRNA expression of cyclin D1, p300 and TERT; **E** Western blot was applied to analysis the protein expression of cyclin D1, p300 and TERT. β-actin was used as internal control. *p* values were calculated by one-way ANOVA in multiple groups and two-sided t-test between two groups. *p < 0.05; **p < 0.01; ***p < 0.001; ****p < 0.0001; ns means* p* > 0.05

As the systemic therapy, it is unknown whether EPCs intravenous injection can attenuate multi-organ senescence in COPD mice. To investigate the effect of EPCs therapy on multi-organ aging, the activity of SA-gal was examined in heart, liver, spleen, and kidney tissues. The results displayed an increased positivity of SA-gal staining in all tissues in COPD, COPD+EPCs and COPD+P5091 groups when compared with control group, and decreased positivity in COPD+EPCs and COPD+P5091 groups when compared with COPD group (Figs. 3, 4, 5, 6A). To explore the effect of EPCs therapy on these organs aging from multiple perspectives, in each tissue we examined the expression of aging-associated indicators, including cyclin D1, TERT and p16 (INK4a). The results of qRT-PCR showed that the relative mRNA expression of cyclin D1 and TERT were significantly downregulated (p < 0.001), while the mRNA expression of p16 (INK4a) was significantly upregulated (p < 0.001) in each tissue of COPD group when compared with control group. Compared with COPD group, cyclin D1 and TERT mRNA expression were increased in COPD+EPCs and COPD+P5091 groups (p < 0.05), but lower than the control group. The mRNA expression of p16 (INK4a) in COPD+EPCs and COPD+P5091 groups were decreased (p < 0.001) when compared with COPD group, but still higher than the control group (Figs. 3, 4, 5, 6B–D). In terms of translation level, the results of Western blotting were consistent with the trend of qRT-PCR. Results showed that the protein expression of cyclin D1 and TERT decreased in COPD group when compared with the control group (p < 0.0001), while the expression of p16 (INK4a) increased significantly (p < 0.0001). Compared with COPD group, the expressions of cyclin D1 and TERT were significantly increased in COPD+EPCs and COPD+P5091 groups (p < 0.05), while the expression of p16 (INK4a) was markedly decreased (p < 0.001) (Figs. 3, 4, 5, 6E). However, there was no difference in the mRNA and protein expression of cyclin D1, TERT and p16 (INK4a) between COPD+EPCs and COPD+P5091 groups, indicating that the therapeutic effects of the two interventions were comparable. These results revealed that the multiple organs of COPD mice suffered from senescence. Moreover, EPCs treatment could affect the transcription of aging-related markers by down-regulating the expression of pro-aging elements and up-regulating the expression of anti-aging elements, which affected the translation of aging-related markers in organs of COPD mice, and finally exerted the effect of inhibiting aging in multiple organs of COPD mice. Meanwhile, the inclusion of the P5091 treatment group increased the credibility of the experimental results.

### EPCs therapy inhibited the expression of mRNA and protein of USP7/p300 in multi-organs

**Fig. 7:**
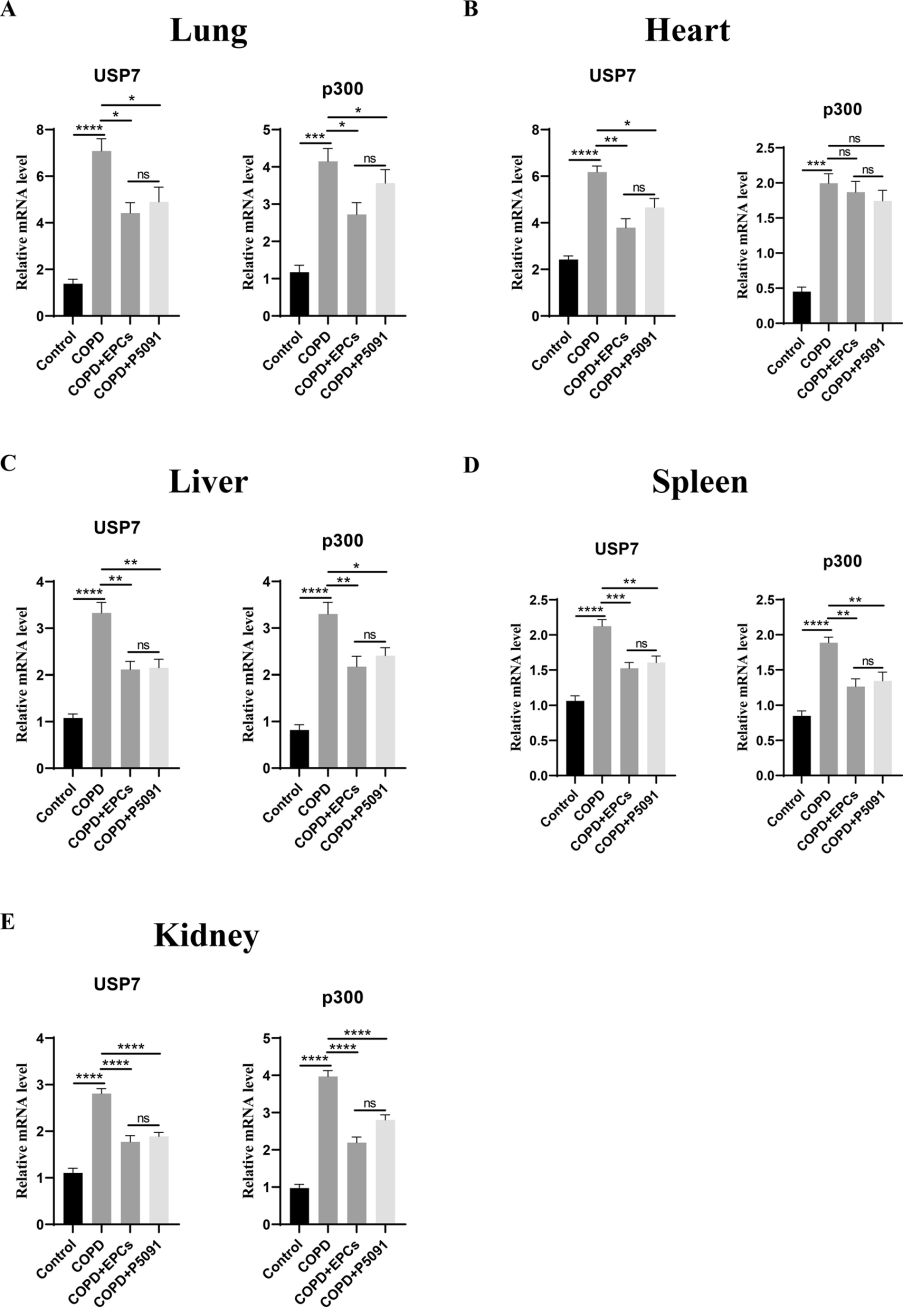
Relative mRNA expression of USP7 and p300 of multiple organs with different interventions. qRT-PCR experiments were used to investigate the relative mRNA expression of USP7 and p300 in lung (**A**), heart (**B**), liver (**C**), spleen (**D**) and kidney (**E**) tissues in control, COPD, COPD + EPCs and COPD + P5091 groups. *p* values were calculated by one-way ANOVA in multiple groups and two-sided t-test between two groups. *p < 0.05; **p < 0.01; ***p < 0.001; ****p < 0.0001; ns means* p* > 0.05

**Fig. 8:**
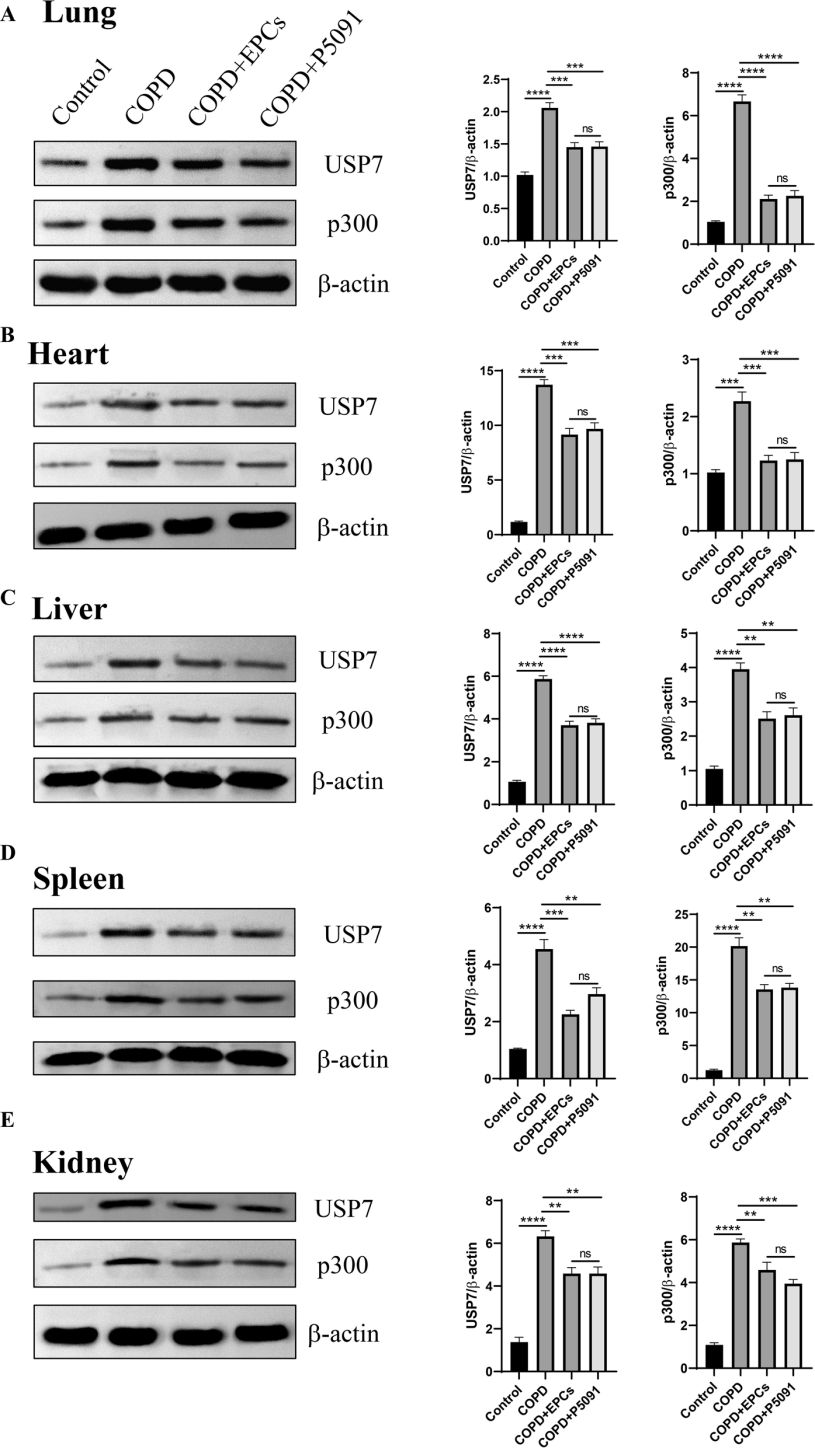
Protein expression of USP7 and p300 of multiple organs with different treatments. The representative pictures (left) and quantitative analysis (right). Western blotting was performed to analyze the protein expression of USP7 and p300 in the lung (**A**), heart (**B**), liver (**C**), spleen (**D**) and kidney (**E**) in the control, COPD, COPD+EPCs and COPD+P5091 groups.* p* values were calculated by one-way ANOVA in multiple groups and two-sided t-test between two groups. *p < 0.05; **p < 0.01; ***p < 0.001; ****p < 0.0001; ns means* p* > 0.05

According to our previous study, aging of EPCs in COPD mice was induced by activation of the USP7/p300 pathway [32]. To investigate the mechanism by which EPCs systemic therapy alleviated aging in multiple organs, we detected the expression of USP7 and p300 in lung, heart, liver, spleen and kidney tissues. qRT-PCR results showed that in multiple tissues, the relative mRNA expression of USP7 and p300 was significantly upregulated in COPD group when compared with control group (p < 0.001). The mRNA expression of USP7 and p300 was markedly downregulated in COPD+EPCs and COPD+P5091 groups when compared with the COPD group (p < 0.05) (Fig. 7). Furthermore, we obtained the same results of western blotting as qRT-PCR. Specifically, in these tissues, the protein expression of USP7 and p300 was remarkably increased in COPD group when compared with control group (p < 0.01). The protein expression of USP7 and p300 was decreased in COPD+EPCs and COPD+P5091 groups when compared with COPD group (p < 0.01) (Fig. 8). The above results suggested that EPCs could suppress the transcription of USP7 and p300, which influenced the protein expression of USP7/p300 pathway. It also revealed that the systemic therapy of EPCs could alleviate the aging of multiple organs by down-regulating the mRNA and protein expression of USP7/p300 pathway in each organ of COPD mice, and then exerted therapeutic effects in COPD mice.

## Discussion

**Fig. 9:**
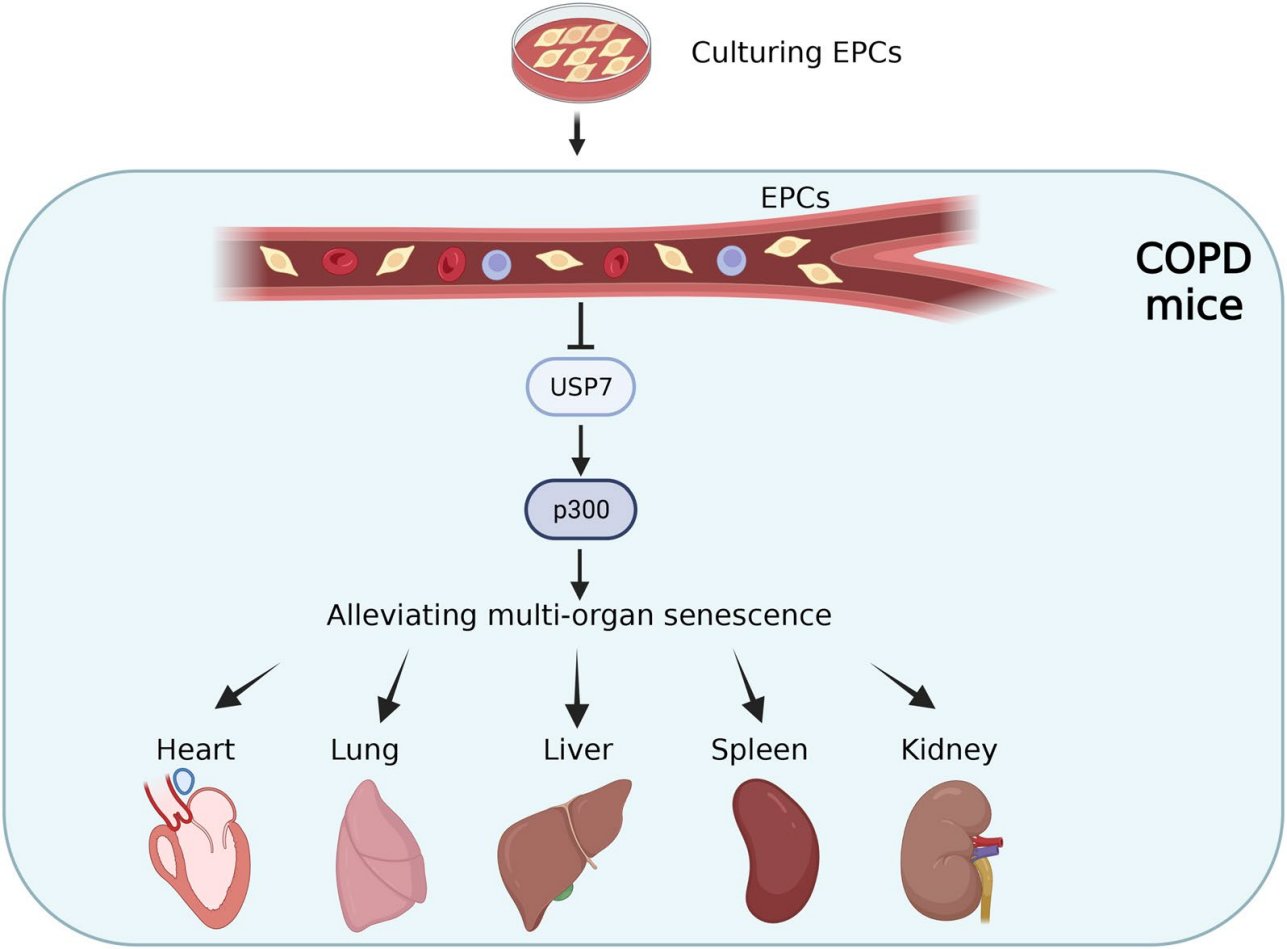
This study demonstrated for the first time that EPCs systemic administration can alleviate the senescence of lung, heart, liver, spleen and kidney caused by COPD by inhibiting USP7/p300 pathway. We aim to provide the possibility for future clinical application of EPCs therapeutic strategy

Here, results showed that compared with control group, there was increased SA-gal activity, upregulated protein and mRNA expression of p16, and downregulated protein and mRNA expression of cyclin D1 and TERT in lung, heart, liver, spleen and kidney tissues of CS-induced COPD mice. When compared with COPD group, SA-gal activity was decreased, protein and mRNA expression of p16 was down-regulated, and protein and mRNA expression of cyclin D1 and TERT were up-regulated of multiple organs in the EPCs system intervention. These demonstrated that systemic EPCs administration significantly attenuated the aging of multiple organs in COPD mice. Mechanistically, protein and mRNA expression of USP7 and p300 were increased in multiple organs in the COPD group compared to controls, whereas intravenous injection of EPCs down-regulated protein and mRNA expression of USP7 and p300 in multiple organs. These suggest that EPCs may down-regulate the USP7/p300 pathway to alleviate multi-organ aging in COPD mice (Fig. 9).

In this study, we used CS-induced COPD mouse model, which can reflect the pathological features of human COPD, such as chronic lung inflammation, pulmonary hypertension, airway remodeling, emphysema, and impaired lung function [33]. Therefore, the experimental results obtained from this model are also more easily available for clinical treatment. This study showed that in COPD mice, the histomorphology of lung tissue was significantly altered, while other organs including heart, liver, kidney and spleen maintained normal structure and histomorphology. Nevertheless, not only in lung tissue, but also in other organs, the mRNA and protein expressions of the specific markers of cell senescence SA-gal and p16(INK4a) were significantly up-regulated, while the expression of cyclin D1 and TERT were significantly down-regulated. These results illustrated the presence of senescence in multiple organs of COPD mice, which is consistent with our previous study [15]. It also indicated the possibility of aging-induced multiple organ dysfunction in COPD mice, which is in accordance with the clinical manifestation of pulmonary dysfunction in COPD patients, followed by other organs dysfunction.

Cellular senescence is a process inducing permanent cell cycle arrest in response to various damage stresses [34]. Nuclear DNA damage is a common initial factor in senescence. DNA damage response causes cell cycle arrest by promoting the activation of p53, which triggers cellular senescence [35]. In addition to cell cycle arrest, senescence mechanisms include telomeres shortening and damage, oxidative damage, mitochondrial damage, and up-regulated anti-apoptotic proteins to promote apoptosis resistance. Furthermore, senescent cells exhibit complex senescence-associated secretory phenotypes (SASPs), such as secretion of pro-inflammatory cytokines and pro-fibrotic mediators, contributing to systemic inflammation and fibrosis of tissues and organs, as well as metabolic changes such as increased SA-gal [36, 37]. Lysosomes accumulated in cells exposed to aging-inducing pressures, and accordingly, the activity of lysosomal SA-gal was significantly increased. However, this specific marker also had limitations, and it was preferable to combine with other markers [38]. Cyclin D1 promoted cell proliferation from G1 to S phase by interacting with cyclin-dependent kinase (CDK) 4/6. Down-regulated cyclin D1 induced cell cycle arrest, leading to cellular senescence [39]. p16 (INK4a) competed with cyclin D1 to bind CDK4/6, causing cell cycle arrest in G1 phase and promoting cells aging. Currently, p16 (INK4a) is considered as one of the senescence markers [40, 41]. TERT, a core component of telomerase catalytic activity, promotes replication and lengthening of telomeric DNA to stabilize the length of chromosomal telomeric DNA, thereby preventing cellular senescence [42, 43].

Aging is a complex and multi-system process in which the biological aging of one organ system selectively affects multiple other systems through specific aging pathways. Controlling gender and physical age, organ age remains a high-risk factor for mortality, especially pulmonary age, preceded by age of renal, hepatic, metabolic, immune, and cardiovascular systems [44]. Research has shown that the aging immune system can drive aging-related changes in other systems [45]. COPD is regarded as a premature-aging pulmonary disease with pathogenesis including chronic inflammation, immune imbalance, and oxidative damage [46]. Pulmonary aging is a significant risk factor for elderly lung disease and is mainly caused by telomere dysfunction-mediated accelerated replicative senescence of pulmonary epithelial stem cells and EPCs [47, 48]. Due to the amount of energy requirements for sustained cardiac contraction, mitochondrial dysfunction and its by-product reactive oxygen species are crucial causes of cardiac senescence [49]. Our previous studies have shown that senescence and depletion of EPCs are present in COPD mice [50]. Increased senescent cells and SASPs in the vascular system result in impaired angiogenesis, chronic inflammation, pathological remodeling of the extracellular matrix and barrier disruption, leading to multiple tissue aging [51].

Normally, stem/progenitor cells are in a resting state, but when the body is stimulated by pathological signals such as injury or aging, stem cells are activated, migrate to the injury site and differentiate into functional cells to replace the damaged or senescent cells, so that the body is in a state of dynamic balance between aging and organ regeneration [52]. EPCs, precursor cells of endothelial cells, can promote vascular regeneration and thus restore blood perfusion and nutrient supply [53]. However, with growing age, the activity of tissue-specific EPCs gradually decreases, together with aggravated senescence of COPD, which makes the rate of regeneration in tissues and organs weaker than that of senescence, and eventually organs or individuals show symptoms of senescence. We have demonstrated the presence of EPCs senescence and depletion in COPD mice [54]. Therefore, stem cell senescence is the underlying cause of bodies aging, and stem cell therapy could contribute to promote the repair of damaged tissues to counteract aging. In this study, we investigated the effects of EPCs systemic therapy on multiple organs aging in COPD mice and used P5091, which has been shown to be effective [32], as the positive control. The results showed that EPCs systemic therapy failed to reverse morphological changes in lung tissue, consistent with previous studies [15]. Intratracheal administration of EPCs was shown to only mildly attenuate histomorphology changes of emphysema in COPD mice, with non-significant changes in DI and MLI [55]. Typically, alterations at the intracellular molecular level precede the morphological changes in the tissue. Therefore, we examined the expression of aging parameters after EPCs intervention. The results showed that EPCs therapy significantly reduced the senescence of lung tissues, and also revealed that EPCs therapy had a protective effect on COPD mice to delay the progression of the disease. Whereas EPCs systemic therapy could not recover the normal morphology of lung tissue, the following reasons may be given: (i) EPCs were administered intravenously in this study, and the efficacy on lung may be weaker than that of direct intratracheal administration; (ii) EPCs systemic therapy was performed after modeling 90 days in this study to simulate clinical COPD patients. The timing of intervention may be late. Irreversible pathological changes in lung tissue had already developed; (iii) EPCs were given only three times in COPD mice. We prudently hypothesized that increasing the times of EPCs treatment would hopefully alleviate the change of morphology in lung caused by COPD. In addition, by intravenous injection, EPCs migrated into multiple organs and functioned as systematic therapy. The results showed that EPCs systemic therapy significantly reduced the senescence of heart, liver, kidney and spleen tissues. This study demonstrated for the first time that EPCs systemic therapy could reduce the aging of multiple organs in COPD mice, delaying the disease progression.

Our previous findings suggested that cigarette smoke extract (CSE) induced EPCs senescence by up-regulating USP7 to activate p300-p53/p21 pathway [32]. Ubiquitination-induced protein degradation is the specific degradation of multiple types of impaired proteins with ubiquitin labels, whereas deubiquitinating enzymes can remove ubiquitin chains from specific proteins to reverse ubiquitination-mediated protein degradation. The interaction between ubiquitinating and deubiquitinating enzymes is necessary to sustain protein homeostasis [56]. USP7 is a deubiquitinating enzyme present in all eukaryotic cells and is indirectly involved in the development and repair of DNA damage [57]. The present study showed that compared to control, mRNA and protein expression of USP7 was significantly upregulated in multiple organs of COPD mice leading to imbalance of ubiquitination and deubiquitylation. It has been demonstrated that suppression of USP7 by genetic depletion selectively induced apoptosis of senescent cells via restoration of p53 activity [31]. Histone acetyltransferase p300 is one of the substrates of USP7, which regulated cell growth and proliferation by acetylating histones or other proteins. Upregulated p300 could lead to senescence-like changes in endothelial cells [32, 58]. We also confirmed that mRNA and protein expression of p300 was upregulated in multiple organs of COPD mice. Because of the intimate relationship between the USP7 pathway and aging, USP7 has been considered as a potential target for aging treatment. Studies have shown that USP7 inhibitors effectively eliminated senescent cells and inhibited doxorubicin-induced aging-related secretory phenotypes in mice [31]. Taken together, we hypothesized that EPCs systemic therapy attenuates senescence in multiple organs by inhibiting the USP7/p300 pathway. The present study demonstrated that EPCs therapy down-regulated the mRNA and protein expression of USP7 and p300 in lung, heart, liver, kidney and spleen tissues. Even in lung and kidney tissues, the downregulation of mRNA expression of USP7 and p300 induced by EPCs therapy was more pronounced than that of USP7 inhibitor P5091. These results confirmed that the attenuation of aging in COPD mice by EPCs was achieved through inhibition of USP7/p300 pathway.

This study has some limitations. Firstly, in the study, only one COPD modeling method was used, without repeating by other COPD models such as elastase-induced models. Secondly, only the mechanism of the USP7/p300 pathway of EPCs treatment in COPD has been studied, and more potential mechanisms of EPCs therapy in COPD have not been thoroughly explored. This is a direction we need to continue to study in depth in the future. Thirdly, this study focused on large solid organs, and we ignored the changes in some important organs, such as the adrenal gland.

## Conclusion

This study demonstrated for the first time that EPCs applied intravenously can reduce the aging of lung, heart, liver, spleen and kidney caused by COPD, and briefly explored the mechanisms involved, providing the possibility for future clinical application of EPCs therapeutic strategy. In-depth investigation of the mechanism of EPCs therapy in COPD is needed in future.

### Supplementary Information

**Additional file 1: Figure S1. **The identification of EPCs. Representative images of immunofluorescence staining of EPCs. **A** EPCs were incubated with Dil-Ac-LDL after 8 days of culture, ×400; **B** EPCs were incubated with FITC-UEA -1 after 8 days of culture, ×400; **C** EPCs were incubated with DAPI after 8 days of culture, ×400; **D** Overlap of two dyes Dil-Ac-LDL and FITC-UEA -1, ×400. The white arrows point to EPCs. Scale represents 100 μm.  

**Additional file 2: Table S1.** List of primer sequences.

**Additional file 3: Figure S2.** HE staining of heart, liver, spleen and kidney tissues. Representative images of HE staining of heart (**A**), liver (**B**), spleen (**C**), and kidney (D) tissue in the control (i), COPD (ii), COPD+EPCs (iii), and COPD+P5091 (iv) groups. Scale represents 50 μm.

## Data Availability

All data generated or analyzed during this study are included in this published article. The data that support the findings of this study are available from the corresponding author upon reasonable request.

## Abbreviations

COPD: Chronic obstructive pulmonary disease
EPCs: Endothelial progenitor cells
USP7: Ubiquitin specific protease 7
CS: Cigarette smoke
MSCs: Mesenchymal stromal stem cells
MLI: Mean lung interlining interval
DI: Destruction index
PBS: Phosphate buffered saline
HE: Hematoxylin–eosin
SA-gal: Senescence-associated β-galactosidase
TBST: Tris-buffered-saline with Tween-20
qRT-PCR: Quantitative real-time PCR assay
TERT: Telomerase reverse transcriptase
ANOVA: Analysis of Variance
CSE: Cigarette smoke extract
CDK: Cyclin-dependent kinase
SASPs: Senescence-associated secretory phenotypes
