# Supplementary figures and images for: Endothelial progenitor cells systemic administration alleviates multi-organ senescence by down-regulating USP7/p300 pathway in chronic obstructive pulmonary disease
Source: J Transl Med. 2023 Dec 6;21:881. doi: 10.1186/s12967-023-04735-x (PMC10699081; doi:10.1186/s12967-023-04735-x)

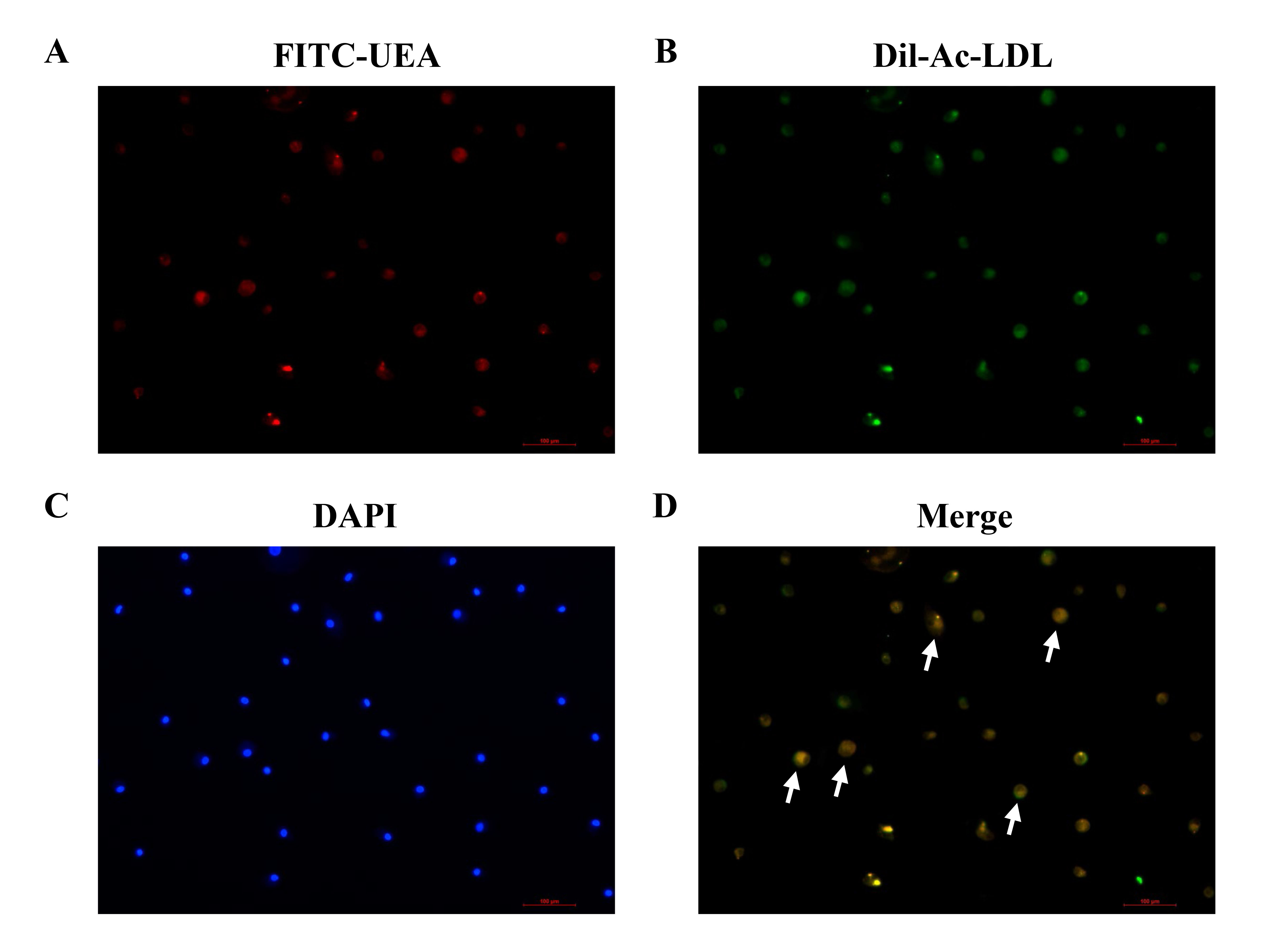

Supplement: Supplementary file 1 — Additional file 1: Figure S1. The identification of EPCs. Representative images of immunofluorescence staining of EPCs. A EPCs were incubated with Dil-Ac-LDL after 8 days of culture, ×400; B EPCs were incubated with FITC-UEA -1 after 8 days of culture, ×400; C EPCs were incubated with DAPI after 8 days of culture, ×400; D Overlap of two dyes Dil-Ac-LDL and FITC-UEA -1, ×400. The white arrows point to EPCs. Scale represents 100 μm. [file 12967_2023_4735_MOESM1_ESM.jpg]

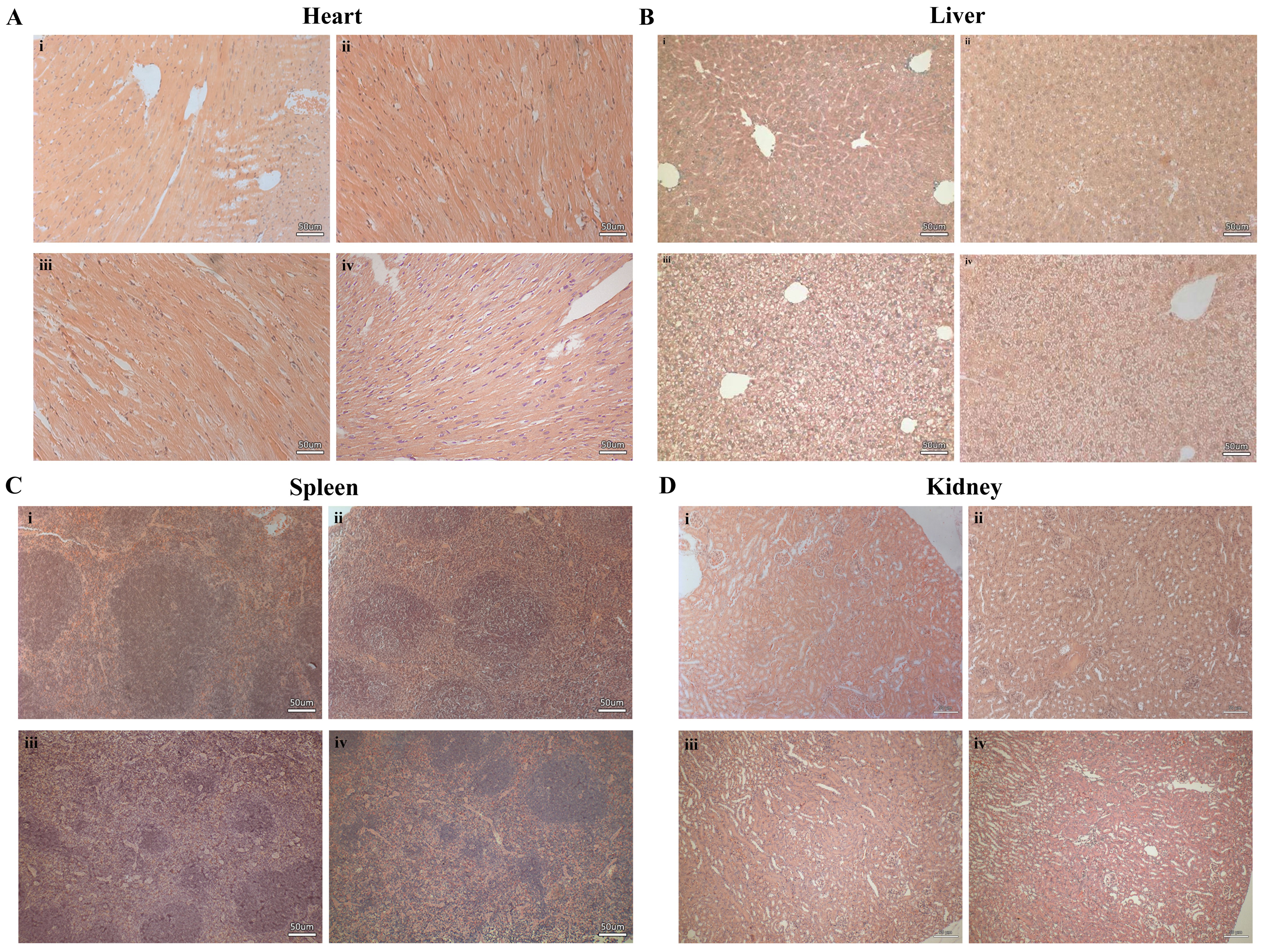

Supplement: Supplementary file 3 — Additional file 3: Figure S2. HE staining of heart, liver, spleen and kidney tissues. Representative images of HE staining of heart (A), liver (B), spleen (C), and kidney (D) tissue in the control (i), COPD (ii), COPD+EPCs (iii), and COPD+P5091 (iv) groups. Scale represents 50 μm. [file 12967_2023_4735_MOESM3_ESM.jpg]
