# Supplementary material for: Endothelial progenitor cells systemic administration alleviates multi-organ senescence by down-regulating USP7/p300 pathway in chronic obstructive pulmonary disease
Source: J Transl Med. 2023 Dec 6;21:881. doi: 10.1186/s12967-023-04735-x (PMC10699081; doi:10.1186/s12967-023-04735-x)
Supplement: Supplementary file 2 — Additional file 2: Table S1. List of primer sequences. [file 12967_2023_4735_MOESM2_ESM.pdf]

Additional file 2: Table S1 List of primer sequences

Primer name	Forward primer	Reverse primer
Mouse		
USP7	CAATGAGGACTGCTCACCCA	AGTCTGAGCAACCCCAACAA
P16 (INK4a)	CAGAGCTAAATCCGGCCTCA	CAGTTTCTCATGCCATTCCT
cyclin D1	TCAAGTGTGACCCGGACTG	ATGTCCACATCTCGCACGTC
TERT	CTGTGCCTACCAGGGGAGAT	GGCCTTGAGCCCAGAAAGAT
p300	AGGAGGAAGAACAGCCAAGC	AGGGATTCCTAGAAGCTGCG
β-actin	ACATCCGTAAAGACCTCTATGCC	TACTCCTGCTTGCTGATCCAC
